# The theoretical and practical difficulties of evaluating a community-based ‘whole systems’ obesity prevention intervention: a research team’s critical reflection

**DOI:** 10.1177/17579139231195700

**Published:** 2023-09-09

**Authors:** EW Gadsby, S Hotham, R Merritt

**Affiliations:** Faculty of Health Sciences and Sport, University of Stirling, Stirling FK9 4LA, UK; Centre for Health Services Studies, University of Kent, Canterbury, UK; Centre for Health Services Studies, University of Kent, Canterbury, UK; Centre for Health Services Studies, University of Kent, Canterbury, UK

**Keywords:** evaluability assessment, obesity, community-based interventions, complexity, Soft Systems Methodology

## Abstract

**Aims:**

This article critically discusses the purpose, pragmatics and politics of conducting commissioned evaluations on behalf of public sector organisations by drawing on the experience of evaluating a community-based ‘whole systems’ obesity prevention intervention for an English local council.

**Methods:**

The study presented in this article incorporated two approaches: an evaluability assessment that interrogated the theoretical and practical difficulties of evaluating the intervention in a non-political way, and a retrospective analysis using Soft Systems Methodology that interrogated the more political difficulties of conducting such an evaluation in the ‘real world’. The information and insights that enabled these reflections came from over 3 years of working closely with the programme team, attending and participating in stakeholder events and meetings, presenting to the Council’s Scrutiny Committee meetings, four interviews with the programme manager, and multiple face-to-face group meetings, email exchanges and telephone conversations.

**Results:**

The study reveals and analyses three key inter-related challenges that arose during the evaluation of the ‘whole systems’ obesity prevention intervention: the programme’s evaluability, the evaluation purpose, and the nature, role and quality of evidence.

**Conclusions:**

The evaluability assessment was important for defining the programme’s theoretical and practical evaluability, and the retrospective analysis using Soft Systems Methodology enabled a greater understanding of the political tensions that existed. Key learning points related to the challenges that arose during this evaluation have broad applicability.

## Introduction

Local councils in England are responsible for public health services and improving the health of their local population.^1^ Many are faced with complex issues such as obesity and physical inactivity, as well as persistent inequalities. They are also expected to ‘do more with less’, as their populations steadily increase and budgets are squeezed.^2,3^ In this context, it is difficult for decision-makers to make sense of and know how best to contribute towards improvement of their population health situation. In the midst of this uncertainty, decision-makers look for evidence – particularly in the form of local programme evaluations – to guide the often messy process of strategy making.

To inform future decisions on their child obesity strategy, a local council in England designed and implemented a 3-year (2015–2018) community-based intervention within a particular ward in the borough. They also commissioned a contractor to work with them to conduct a robust and independent evaluation.

### The intervention

The intervention was a community-based programme that aimed to prevent overweight and obesity in children through a system-wide, multistakeholder approach. The intention was to mobilise and involve everybody who has a stake in the community (including children and families, childcare settings, the voluntary sector, private businesses, politicians, council departments, etc.); to enable local stakeholders to implement effective and sustainable activities to promote healthy lifestyles; and to create a local environment that better supports healthy lifestyle choices. It sought to raise awareness and knowledge of healthy eating and physical activity, as well as enable micro-environmental behaviour changes, through social marketing campaigns. Each campaign incorporated information dissemination, training opportunities for people working with children and families, working with council departments and local agencies, and development activities including a grant scheme, local events and other ad hoc support for local groups and organisations.

### The evaluation

The aim of the evaluation was ‘to assess the impact of the system-wide approach on the key areas defined by the specific themes’ (as stated in the service specification). Ultimately, the commissioners expected that changes in awareness, knowledge, skills and behaviours of people who influence children’s environments and of children themselves, would translate into an increase in the percentage of children with a healthy weight. However, as discussed later in this article, the evaluation’s purpose – and consequent implications for design and conduct–warrants further critical reflection.

### Purpose of this article

This article draws on the experiences of the evaluation team and critically explores the complexities of evaluating multistrategy, community-based approaches to obesity prevention on behalf of a public sector commissioner. It acknowledges the theoretical and practical difficulties of evaluating complex interventions, which are now well-rehearsed in the evaluation literature (see below). It examines these in relation to the intervention in question and describes the findings of an Evaluability Assessment conducted at the start of the evaluation. From a reflective viewpoint (after the completion of the evaluation), it then goes on to interrogate the more pragmatic and political difficulties of conducting such evaluations as a commissioned exercise, using systems thinking. The article reveals and analyses three key inter-related challenges that arose during the evaluation: the programme’s evaluability, the evaluation purpose, and the nature, role and quality of evidence. Finally, it proposes key learning points related to these challenges that will be common to many situations.

## Background

### Child obesity and whole systems approaches

Childhood obesity is recognised as one of the most serious health challenges of the 21st century.^4^ The inequalities in childhood obesity are compelling and the widening of the ‘obesity gap’ over the past decade has prompted calls for more focused efforts to target those most at risk.^5^ An interest in ‘whole systems approaches’ has emerged from a recognition of the complexity of obesity causation and prevention and a frustration with the lack of success of efforts over the last few decades.^6,7^ Whole systems approaches seek to link together many of the influencing factors on obesity in a coordinated and integrated effort, across multiple sectors, to bring about change. Informed by complexity theory, their characteristics include the recognition of nonlinearity, dynamic interconnectedness between causes and influences, adaptive agents, networks and relationships, and the importance of understanding how the whole system can be ‘more than the sum of its parts’.^8^ However, the language, theory and practice of whole systems approaches – certainly within the public health field – is still young. There is no shared understanding of how best to apply systems thinking, what a whole systems approach to obesity looks like in practice, or of what is most likely to work and have meaning in systems at different levels. Little is known about the key mechanisms of change; they are likely to be many, as well as time- and context-specific. Robust and relevant evidence is needed to help identify and implement effective whole systems responses. But the challenges of producing such evidence in this area has prompted a call for a radical re-think around the traditional biases in public health research funding, activity and publication, as well as much discussion regarding methodologies.^9^

### Evaluating whole systems approaches

The challenges of evaluating complex, systems-wide public health interventions are now well-rehearsed in the literature.^10–14^ They relate to the presence of multiple programme components (with the belief that a certain synergy will be achieved among them), action at multiple levels (and the notion that there is interaction among those levels), the importance of context, the flexible and evolving nature of the interventions, the breadth and often long-term nature of the range of outcomes being pursued, and the absence of appropriate control groups for comparison purposes.^15,16^ It is unsurprising, given these challenges, that there is a paucity of evidence on the identification, implementation and evaluation of effective community-wide programmes for obesity prevention.^17^

Theory-based approaches have demonstrated promise in helping evaluators to come to terms with the inherent complexity of certain types of interventions and to overcome the limitations of experimental evaluation designs.^15^ Theory-of-change and realist evaluations are two prominent categories of theory-based approaches that have been used to evaluate health improvement interventions. While they are distinctly different approaches, both emphasise the importance of context in understanding how complex programmes can lead to changes in outcomes, and both are concerned with understanding the theory of an initiative, and with using that theory to inform the evaluation’s purpose, focus and methods.^18^ As limitations and challenges of these approaches have been identified, and experience progressed, evaluation practice has continued to evolve. Some researchers have, quite naturally, begun to draw on complexity theory to add value to theory-based approaches.^11,13,14,19–22^

### Conducting commissioned ‘real world’ evaluations

In conducting an evaluation for a local authority commissioner, evaluators are thrown into the messy, poorly controlled situation of what Robson calls ‘real world research’.^23^ Evaluations operate within political constraints, and are politically articulated. For the commissioners, they are an important means through which local decision-makers develop and adapt their approaches to health improvement. They are also important in the context of council officers’ and elected members’ concerns with accountability to others. They must frequently defend their chosen course of action and their professional or organisational credibility to the public (their local electorate), to councillors and officers across the council, and to other stakeholders and external funders. Evaluation activities can be important, then, in managing some of the reputational risks that arise, particularly from developmental work, by demonstrating that a programme was effective in the face of potential criticism.^24^

Evaluators must make judgements that could have far-reaching consequences; a poor evaluation report, for example, may lead to termination of a particular programme or services.^25^ Academic evaluators are also driven by the need to publish in peer-reviewed journals, and by codes of academic and professional integrity. Options are often severely limited by ‘only-just-enough’ budgets (particularly when contracts are won through a competitive tendering process), and evaluators find themselves walking a tight-rope between ‘quick and dirty’ forms of evaluation and ‘evaluation research’ that profits from a principled systematic approach and is concerned with generating new knowledge.^23^ The sensitive and political nature of evaluation demands careful, strategic thinking regarding the purpose, design and conduct of the research. The remainder of this article describes the strategic thinking of the authors regarding the evaluation of the child obesity prevention intervention. The purpose is to draw out learning, based on our experience, for evaluators in similar situations.

## Methods: Strategic Thinking Regarding The Purpose, Design And Conduct Of Evaluation Research

The study presented here incorporates two approaches: (1) an evaluability assessment that interrogated the theoretical and practical difficulties of evaluating this intervention in a nonpolitical way; (2) an analysis using Soft Systems Methodology that interrogated the more political difficulties of conducting such an evaluation in the ‘real world’, as a commissioned exercise.

The evaluation team (from University of Kent) were contracted prior to the initial launch of the intervention and worked closely with the programme team over a 9-month period to understand the programme design, the underlying programme model and opportunities for useful evaluation. The programme team provided detailed baseline data, vision and mission statements, project plans/descriptions, and written goals and objectives. The programme’s Theory of Change was elicited and clarified through discussion with the programme team. Through this process, assumptions were made explicit and evidence/theories supporting (or undermining) the Theory of Change were articulated. To assist in the planning of the evaluation, and to help explain and rationalise the evaluation design, the systematic approach of an evaluability assessment (EA) was adopted.^26,27^

**Figure 1:**
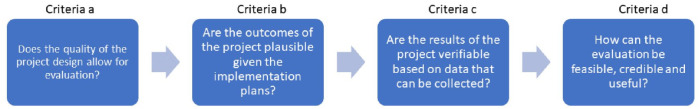
Key criteria considered in the evaluability assessment

The EA engaged the commissioners in considering evaluation challenges and limitations. A logic model was developed and refined in an iterative process. Discussions with programme staff tested, refined and further developed this logic model and helped the team to understand the proposed programme reality. Data needs were identified and reviewed and considered in relation to the logic model. Evaluation and subject matter expertise were then employed to form opinions regarding evaluability and the feasibility of alternative evaluation designs, based on key criteria adapted from an existing EA template:^28^ (1) the quality of the project purpose; (2) the quality of expected outputs; (3) the availability of baseline and monitoring data; and (4) the feasibility of attribution (see Figure 1). The findings of the EA are summarised below under ‘The programme’s evaluability’.

Throughout the evaluation, problematic issues associated with conducting commissioned ‘real world’ evaluations started to emerge. To think strategically about the challenges of conducting this evaluation (and others like it), the authors conducted a retrospective situation analysis using the general principles and key elements of Soft Systems Methodology (SSM).^29^ This organised, action-oriented process of inquiry helped the team to explore the situation in a holistic and pluralistic way, using models as intellectual devices. The methodology was used to reflect on the conduct and complexity of the *evaluation*, rather than to map the *programme* complexity (which had already been explored using the EA). Specifically, the situation was described and understood through the building of a ‘rich picture’ that aimed to capture, informally, the main entities, structures and viewpoints in the situation, the processes going on, and recognised issues. The structured process of SSM was used to inquire into the roles, norms and values of ‘client’, ‘practitioner’ and ‘issue owner’, to surface multiple worldviews, and to explore how power was expressed in the situation. While this was an introspective exercise, the information and insights that enabled this process came from over 3 years of working closely with the programme team, attending and participating in stakeholder events and meetings, presenting to the Council’s Scrutiny Committee meetings, four interviews with the programme manager, and multiple face-to-face group meetings, email exchanges and telephone conversations. Research notes were recorded for all meetings and conversations, and a research diary was maintained throughout, recording the evaluation team’s reflections and thoughts. The process of constructing a conceptual model of the team’s ‘purposeful activity’ helped to identify learning for dealing with challenges related to the evaluation purpose, and the nature, role and quality of evidence.

## The Programme’s Evaluability

### Criteria 1: the nature of the project purpose

**Figure 2:**
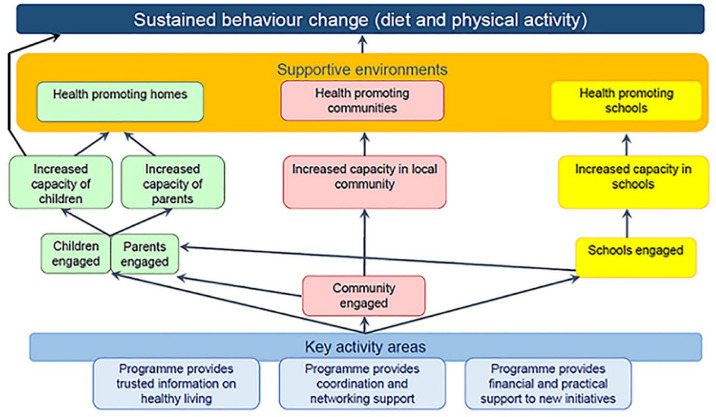
Theory of change diagram

This criterion examined the extent to which the quality of the programme design allowed for evaluation in principle. The programme was established to target resources on a geographical community (an inner-city electoral ward) that had relatively high levels of deprivation and obesity compared with local and regional averages. The theory was that by engaging the whole community and stakeholders within the ward and across the council in a geographically focused initiative, locally appropriate and co-developed activities would be designed and delivered to raise awareness and understanding of the issues (in relation to healthy diet and physical activity), and encourage and support behaviour change among children and their families. The intervention aimed to engage with those with a role in shaping the local environments in which children live, learn and play: community partners (including schools, local businesses, service providers, etc.), parents and children. The Theory of Change is presented in Figure 2. Inputs included a full-time programme manager, support from a communications officer, and a modest programme budget. With this, the intention was to provide trusted information on healthy eating and activity, coordination and networking support for partners, and financial and practical support to new initiatives that would help to support the programme’s aim. Most of the activities were geared towards the community partners, and included engagement events, workshops, training sessions, regular communications and access to funding via a grant scheme. Interim outcomes were expected to be changes in home, school and neighbourhood environments to better support children’s healthy eating and activity, and changes in children’s behaviours in relation to the six dietary and physical activity themes (such as swapping nutrient poor snacks for healthier alternatives, increasing fruit and vegetable consumption, decreasing screen time, and increasing active play).

The justification of the programme was realistic and based on a sound understanding of the local situation, a substantive review of existing obesity prevention interventions and international evidence on effective ways to prevent childhood obesity. The programme was consistent with the recommendation that attempts to influence people’s behaviour should be aimed at changing both physical (or sedentary) activity and diet or healthy eating, and comprise multiple, potentially interacting methods of changing behaviour.^30^ However, the programme theory was understandably complex, and it was difficult to achieve clarity, realism and shared understanding among the stakeholders around the objectives. The EA concluded that the programme theory was underpinned by many assumptions and that the desired behaviour change outcomes would be dependent on these, as well as many external factors (such as counteracting forces in the meso- and macro-environment). Moreover, the success of one aspect of the intervention (changing attitudes to motivate children and parents) would likely rely on the success of the other (managing the environment so that people have increased opportunities or abilities to undertake the desired behaviours).

### Criteria 2: the quality of expected outcomes

This criterion explored the extent to which the outcomes of the programme were plausible, given the way in which it was to be implemented. The expected long-term outcome was an increase in the proportion of children in the ‘healthy weight’ category, according to BMI (body mass index) centiles. Intermediate outcomes related to changes in behaviours among children, in relation to their own diet and physical activity, and among parents, teachers and community partners, in relation to supporting and encouraging healthy child behaviours. Short-term outcomes were the development of awareness, knowledge and positive attitudes among children, parents and stakeholders towards eating well and moving more, and increased capacity among key agencies and groups working with children to support healthy lifestyles.

The programme was adequately resourced, and the programme team had secured political support for the project and engaged local elected members. However, implementation relied heavily on one full-time programme officer who, in the course of several staff re-organisations, faced an uncertain future. The quality of the expected outputs depended heavily on that programme officer, their engagement with the stakeholders, and the continuity they could provide throughout. Since it was also one programme that interacted with others and with the context in which it sat, it also depended on continued investment by the health and local government commissioners in the broad range of health, social care and well-being services. In a context of financial insecurity, budget cuts and organisational upheaval, this continued investment was not a given.

Parents and children were to be targeted by the intervention both directly (through information provision, community events, regular communications), and indirectly (through the work of the community partners). By engaging all schools, and working through a wide range of partners, these actions were likely to increase awareness/knowledge about healthy eating and physical activity among many children and their parents, and would potentially contribute towards the development of positive attitudes towards eating well and moving more. These changes in behavioural determinants might then contribute towards behaviour change among children. Evidence on the complexity of obesity suggests that it would be a considerable challenge to significantly alter a population’s weight status, particularly within a few years.^31^ While this programme had the potential to contribute towards obesity prevention within its target ward, as part of a wide range of micro, meso and macro-level interventions, it was important to be realistic about its potential to alter the outcome of a system as complex and extensive as that driving the weight status of the populations, especially within a 3-year period. Significant, measurable shifts in population behaviours (where they happen), might be anticipated to take at least 2 to 3 years. The unpredictability and non-linearity of this programme is inherent within its community development approach.^13,32^ It was decided that a strong process evaluation would be essential in order to learn lessons for future implementation plans.

### Criteria 3: the availability of data

This criterion examined whether the results of the programme would be verifiable based on the data that could feasibly be collected. The EA considered it was feasible to collect a broad range of data, from numerous sources, that could track both process and outcomes across the logic model. This would, however, place a time burden on the programme team, who would need to collaborate in the creation and management of a data system. The programme team separately commissioned the collection of BMI data for children in the target community throughout the course of the evaluation, providing an objective indicator of population weight change. The evaluation team were employed from early in the programme’s history for 4 years, so data could be collected intermittently over this time frame, allowing good opportunities for short-and medium-term follow-up. Much of the short- and medium-term outcomes data would be self-reported, which has clear limitations; behaviour data reported by young children should be treated with special caution. Achieving high response rates to parent and stakeholder questionnaires is challenging, and those choosing to respond may exhibit particular characteristics over non-respondents. However, the EA concluded that the careful design of questionnaires, the addition of qualitative data collected through interviews and focus group discussions, and the collection of data at multiple time points to investigate change over time, could help ensure self-report biases are reduced, and add richness and understanding to the data.

### Criteria 4: the feasibility of attribution

This criterion examined the extent to which an evaluation would be feasible, credible and useful. Problems associated with attribution, causation and generalisation are common to most health-promotion initiatives. While long-term objectives would be measurable (BMI is a usable indicator of population overweight), it would be difficult to attribute any change to the specific programme. Short-term objectives and proximal outcomes might be more readily attributable to the programme but would be more problematic to measure; SMART (specific, measurable, achievable, realistic and time-bound) indicators are more difficult to identify where the proximal outcome is related to, for instance, community development or capacity strengthening. The EA concluded that a theory of change approach was needed to go some way towards helping to strengthen the scientific case for attributing change in outcomes to the activities included in the initiative, by specifying at the outset how activities will lead to intermediate and long-term outcomes, and by identifying the contextual conditions that might affect them. In addition, the research evaluation would enable the ‘testing’ of some of the key assumptions underpinning the programme theory, which would contribute valuable knowledge.

The evaluation team found the EA to be extremely useful. It verified that the programme was theoretically sound, but highlighted the assumptions underpinning the programme theory and established a sense of realism related to the longer-term outcomes (criteria 1). It highlighted the value of the process evaluation, in helping the council to learn lessons from the pilot (criteria 2). The possibilities and limitations of data collection were made clear, and reassurance was given that the commissioned evaluation would gather sufficient data, of sufficient quality, to answer the key questions (criteria 3). Finally, the EA helped to justify (and explain the value and limitations of) a theory of change approach for this evaluation (criteria 4). Despite this, however, some stakeholders found the EA report challenging. To reflect on why, we now turn to two issues that emerged from our SSM analysis.

## Evaluation Purpose

While evaluations are typically requested to answer the question ‘Does it work?’, decision-makers and other stakeholders ask many questions about interventions that are not just about effectiveness. Questions might include: How does it work? Will service users be willing or want to take up the service offered? Is it the right service for these people? Are users, providers and other stakeholders satisfied with the service? In recognition of the complexity of social change and health improvement, where public health improvements are achieved through the reshaping of multiple interacting factors through multiple interventions, Rutter et al.^9^ recommend that ‘Instead of asking whether an intervention works to fix a problem, researchers should aim to identify if and how it contributes to reshaping a system in favourable ways’.

Our commissioner required the evaluation to

*‘assess the impact of this system-wide approach on the key areas defined by the specific [dietary and physical activity] themes . . . ultimately, we expect that these changes will translate into an increase in the percentage of children with a healthy weight’ (quoted from the service specification for the evaluation).*

Specific research questions were not posed, although the pilot was intended to inform the potential replication of the intervention in other areas of the borough, and inform wider knowledge around community-based ‘whole place’ obesity interventions.

In our conduct of the EA, evaluation purpose was explored only with the immediate programme team, taking our brief from the service specification produced by them. In our retrospective analysis of the situation using SSM as a guide, we more critically considered the purpose of this evaluation, and the multiple perspectives on this. The analytical process helped to further explore important differences in worldviews related to the design, conduct and usefulness of the evaluation. One perspective holds that it would be possible to objectively measure whether this programme works to help tackle obesity, and from that, make evidence-informed decisions about future spending. However, as already alluded to, the assumptions about linear causal pathways both within the programme and in evidence-informed decision-making are problematic. During the EA processes of programme theory development, identification of indicators, and consideration of design and methods, the evaluation and programme teams sometimes found it difficult to identify the most suitable strategy for evaluation.

From our vantage point at the end of the evaluation, and drawing on the rich picture we had created, a ‘root definition’ was defined to describe our ‘system of interest’:

*The evaluation team’s system, enacted by them for the benefit of the council and for more general advancement in academic knowledge, to evaluate the intervention by means of collecting and analysing a range of information in order to better understand what contribution it makes, within this specific context, to tackling childhood obesity, within a four-year period and with a limited budget, and without placing undue financial or time pressures on either the intervention staff or members of the local community, in the belief that this will provide new knowledge regarding the contribution, implementation and evaluation of community-based approaches to obesity prevention.*

**Figure 3:**
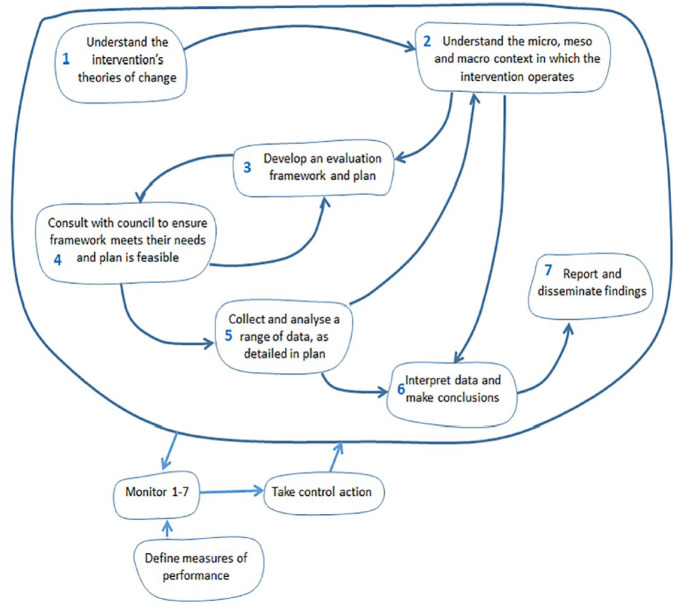
A conceptual model of the system to evaluate the intervention

From this root definition, and from the analyses that contributed to the rich picture, the evaluation team constructed a conceptual model (Figure 3) to identify ways in which the evaluation process might have been improved. Conceptual models, in SSM, are devices that define and link the activities needed to make the required transformation (in this case, helping the council and wider academic and public health communities to better understand the contribution of community-based approaches to obesity prevention). The activity in the operational part of the model should be captured in ‘the magical number 7 ± 2’ activities.^29^ The model is predicated on an understanding of different worldviews. From the evaluation team’s perspective, two standpoints were important: the belief that it is not possible to objectively measure whether this intervention works to tackle childhood obesity, or to distinguish the contribution of this intervention from the contribution of other interventions at different levels; and the belief that in making decisions about spend/investment, the council should consider many issues other than simply ‘does it work’. This is why we were drawn to a theory-based evaluation design. While theory-based approaches to evaluation are becoming more mainstream, their design, potential and limitations are harder to explain to the uninitiated. This leads us on to the final challenge described in this article, relating to evidence.

## The Nature, Role and Quality of Evidence

While the council understood the need for a strong qualitative dimension in the evaluation (as justified in the EA), they were not entirely free from what Schwandt calls the ‘modernist paradigm of reason’.^33^ This is perhaps not surprising, given the present enthusiasm for evidence-based approaches, and the financial squeeze further heightening the pressure to spend money only on ‘what works’. Thus, there were assumptions made about the validity of different forms of knowledge and the value of different types of evidence that presented particular challenges to the evaluation. In our case, there was an overwhelming preoccupation with providing hard, reliable, factual data on children’s dietary and physical activity behaviours. In this evaluation, the most practical and feasible method of accessing information about behaviours from approximately 1000 primary school-aged children was from the children themselves, via questionnaires self-completed in school time. In the absence of any validated survey tools that (1) could be completed by young children themselves and (2) covered the wide range of eating and activity behaviours the intervention sought to change, the evaluation team designed their own survey for this purpose. In designing the questionnaire, the tussle between what was feasible (from an evaluator’s perspective) and what was desired within a normative stance valuing objective, value-free knowledge (from a commissioner’s perspective) was not easy to manage, and compromises were inevitably made. Meanwhile, the qualitative data garnered less interest, and was viewed largely as a supplementary way to explore notions of acceptability and aspects of implementation process. While both the provider and client were satisfied with the final evaluation design and data collection tools, substantial valuable time was taken to arrive at that point.

It was interesting too, that in correspondence and exchanges between the commissioner and evaluation team, there appeared to be a clear assumption that evaluation evidence coming from this pilot programme would allow decision-makers to either adopt this intervention elsewhere in their borough, either in a whole or modified form, or to strike it from their list of intervention options. However, such a rational, linear, evidence-to-policy pathway is neither realistic nor credible.^34^ In this context, evaluators must be mindful of the potential influence (or not) of their evaluation, and take a pragmatic approach to ensuring the immediate usefulness of their work.

## Discussion

From an evaluation team’s point of view, the EA established a sense of realism that was an important basis from which to design the evaluation. Prior to its launch, the programme team had fought hard to secure funding and commitment from elected members and other stakeholders, on the basis that this represented an opportunity to ‘tackle child obesity’ and reduce population overweight. Unrealistic expectations of the programme at the outset meant there was a high risk of determining it inadequate, and therefore of missing crucial opportunities for learning about this kind of approach. They also posed a threat to the evaluation team since stakeholders wanted and expected the evaluation to attribute improvements in long-term outcomes to this intervention. The EA was a thorough and structured way of justifying the final evaluation design, which was theory-led, incorporated a strong process evaluation, relied on bespoke data collection tools for self-reported data, and adopted an ‘action learning’ approach, with annual events for learning and reflection. However, when the EA report was presented to wider stakeholders, this realism was interpreted as overly negative and unconstructive.

While the EA helped to work through issues related to the programme’s theoretical and practical evaluability, it did little to address the apparent tensions related to the context – of designing and conducting this evaluation on behalf of public sector commissioners, with a very limited budget, and with conflicting beliefs/attitudes related to evidence and evidence-based policy-making. The retrospective analysis of the situation using SSM helped the evaluation team to understand some of these aspects in more depth. In particular, it was found that the critical exploration of the evaluation purpose and design achieved in the EA only partially recognised the perspectives of other stakeholders – particularly the elected members.

In a recent paper, Dalkin et al.^19^ explored the compatibility of SSM with realist approaches. In this study, SSM enabled the team to learn retrospectively from their experience. However, an incorporation of SSM into the evaluation design process might have helped the team to deal more effectively and constructively with boundary tensions arising from conflicts between contrasting perspectives. Indeed, in SSM, the user is at the centre of the SSM process – as captured by Checkland and Poulter in the LUMAS model.^29^ The key learning points that emerged from our reflections were:

First, EA is a valuable approach to use in managing expectations and challenging underlying assumptions. High levels of continuous negotiation are required to ensure ‘buy in’ to the approach taken, and to help to ensure the evaluation remains ‘utilisation-focused’.^35^ This might be considered as an embedded approach to research, the relevance and utility of which is increasingly being recognised within efforts to improve complex real-world problems,^36^ but which is difficult to achieve in a commissioned evaluation. An embedded approach contains many elements of action research and ethnography. Thus, researchers need to be equipped to easily navigate the tensions inherent in an embedded approach. Our experience highlighted the importance of building trusting relationships, and the difficulty of doing this where insufficient time has been allowed, and in an organisation/system that is in flux.

Second, multiple perspectives of evaluation purpose will co-exist and should be explicitly acknowledged at the outset. In our example, one measure of effectiveness was the degree to which the evaluation helped the council (and others) to make decisions regarding future spending/investments. This relates back to the role of elected members and their accountability to the public, and their importance as issue owners. The evaluation team were also issue owners, and wanted to produce a robust evaluation that they could be proud of. They therefore needed to maintain a degree of professional independence in order to preserve academic integrity. (This could be seen as a contradiction to the first learning point above – the challenge will be to manage these dialectic tensions). Another measure of ‘system performance’ might include scientific rigour, so issues of reliability and validity should be considered reflexively throughout, with careful considerations of the ‘trustworthiness’ of evaluation findings.^36^ Thurston and Potvin recognise that programme evaluation is an inherently politicised process, rather than a benign technical activity, and argue for a ‘politics of accountability’.^37^ SSM can encourage difference to be understood, and clarity to be achieved regarding the purposes of the evaluation. It can also help to understand the dynamics of power which can shape an evaluation and its dissemination in a variety of ways.

Finally, the needs of the various issues owners should be recognised at different stages, and this should inform the timeline for analysis, reporting and dissemination. In our example, the early communication of ‘quick wins’ (short-term outcomes), and regular feedback on the evaluation process and findings reassured those who were not comfortable with this kind of complex evaluation research. We used ‘evaluation stories’^38^ and annual ‘learning events’ to great effect. Evaluation should be seen as a feedback system between the programme and its environment to facilitate local programme improvement.^39^ The evaluation team therefore should be responsive to changes in the political context, recognising that the demands on the evaluation may change over the lifecycle of the programme. Time and budget place obvious restrictions on evaluation design, but evaluators must also avoid placing undue financial or time burdens on the programme team or members of the local community.

## Conclusions

The analyses described here were valuable in helping to determine the role of and approach taken by the evaluation team, and to retrospectively reflect on the challenges encountered in order to learn from the experience. The EA process considered the programme history, design and operation, its implementation plans, the capacity for data collection, management and analysis, the likelihood that the programme will reach its goals and objectives, and why an evaluation will or will not help the programme and its stakeholders. This pre-evaluation activity helped to develop a pragmatic plan for the evaluation, through the process of collaborating with the programme team to identify the programme logic and make assumptions explicit. Given the rigorous and structured approach, it also helped to construct a solid rationale for the evaluation design. One limitation of this EA was the lack of involvement of a wider range of stakeholders, including members of the target community and elected members in the Council. Even though the programme had been co-developed by all key stakeholders, the EA report produced by the evaluation team challenged some stakeholders’ expectations of the programme and the evaluation. However, among the core programme and evaluation teams, the EA helped to develop a shared mind-set around what might be expected to happen, how that can (and can’t) be measured, and the key areas that the evaluation research should seek to illuminate.

The retrospective analysis of the situation using SSM helped to interrogate further some of the challenges we experienced, to reflect and learn from them. While the problem analysis using SSM was an introspective exercise, conducted retrospectively by the evaluation team, significant strengths of the method lie in its participatory approach. In future, it would be useful to explore the value of conducting an SSM enquiry *during* (or in the early stages of) the evaluation, as a joint endeavour. Nonetheless, analysis presented here helped the team to both reflect on their own approach, and to consider key learning points for others engaging in this type of complex, real-world programme evaluation. It is also recommended that local authorities consider the value of conducting or commissioning an EA before planning a full evaluation, and work closely with any commissioned evaluation teams to engage critically and systemically with the purpose, pragmatics and politics of conducting a proposed evaluation.
